# The Role of the Dynamic Lung Extracellular Matrix Environment on Fibroblast Morphology and Inflammation

**DOI:** 10.3390/cells11020185

**Published:** 2022-01-06

**Authors:** Tillie-Louise Hackett, Noamie R. T. F. Vriesde, May AL-Fouadi, Leila Mostaco-Guidolin, Delaram Maftoun, Aileen Hsieh, Nicole Coxson, Kauna Usman, Don D. Sin, Steve Booth, Emmanuel T. Osei

**Affiliations:** 1 Centre for Heart Lung Innovation, St. Paul’s Hospital, Vancouver, BC V5Z 1M9, Canada; TILLIE.HACKETT@HLI.UBC.CA (T.-L.H.); n.r.t.f.vriesde@student.rug.nl (N.R.T.F.V.); May.Fouadi@hli.ubc.ca (M.A.-F.); dmaftoun@yahoo.com (D.M.); Aileen.Hsieh@hli.ubc.ca (A.H.); Nicole.e.coxson@gmail.com (N.C.); Kauna.Usman@hli.ubc.ca (K.U.); Don.Sin@hli.ubc.ca (D.D.S.); steve.booth@zymeworks.com (S.B.); 2 Department of Anesthesiology, Pharmacology and Therapeutics, University of British Columbia, Vancouver, BC V5Z 1M9, Canada; 3 Department of Systems and Computer Engineering, Carleton University, Ottawa, ON K1S 5B6, Canada; leila@sce.carleton.ca; 4 Department of Medicine, University of British Columbia, Vancouver, BC V5Z 1M9, Canada; 5 Department of Biology, University of British Columbia, Vancouver, BC V5Z 1M9, Canada

**Keywords:** lung, fibroblasts, cell morphology, inflammation, cell death, lung dynamics, fibrosis, collagen

## Abstract

The extracellular matrix (ECM) supports lung tissue architecture and physiology by providing mechanical stability and elastic recoil. Over the last several decades, it has become increasingly clear that the stiffness of the ECM governs many cellular processes, including cell-phenotype and functions during development, healing, and disease. Of all the lung ECM proteins, collagen-I is the most abundant and provides tensile strength. In many fibrotic lung diseases, the expression of collagen is increased which affects the stiffness of the surrounding environment. The goal of this study was to assess the effect on fibroblast morphology, cell death, and inflammation when exposed to 2D and 3D low (0.4 mg/mL) versus high (2.0 mg/mL) collagen-I-matrix environments that model the mechanics of the breathing lung. This study demonstrates that human fetal lung fibroblasts (HFL1), grown in a 3D collagen type-I environment compared to a 2D one, do not form cells with a myofibroblast morphology, express less F-actin stress fibers, exhibit less cell death, and significantly produce less pro-inflammatory IL-6 and IL-8 cytokines. Exposure to mechanical strain to mimic breathing (0.2 Hz) led to the loss of HFL1 fibroblast dendritic extensions as well as F-actin stress fibers within the cell cytoskeleton, but did not influence cytokine production or cell death. This dynamic assay gives researchers the ability to consider the assessment of the mechanodynamic nature of the lung ECM environment in disease-relevant models and the potential of mechano-pharmacology to identify therapeutic targets for treatment.

## 1. Introduction

The extracellular matrix (ECM) supports the tissue architecture of the lung by providing mechanical stability and elastic recoil, which are essential for normal lung physiology [1,2]. This is achieved by the lung ECM being composed of a complex mixture of fibrous proteins, including collagens and fibronectins, which provide tensile strength; elastin molecules, which provide elastic recoil; and adhesive glycoproteins (fibronectin, laminin), glycosaminoglycans (heparin, hyaluronic acid), and proteoglycans (heparin, chondroitin sulfate), which resist compressive forces [3]. In addition to providing structural integrity, the lung ECM microenvironment shapes cell behavior both in health and disease via its molecular composition, concentration, stiffness, and ability to sequester growth factors that can bind directly to the ECM [4,5]. Over the last several decades, it has become increasingly clear that the stiffness of the ECM governs many fundamental cellular processes, including cell adhesion, cytoskeleton, gene expression, migration, polarity, proliferation, and signaling throughout the life span [6,7,8], including embryonic development, organogenesis, angiogenesis, wound healing, ageing, disease, and metastasis. On a tissue level, the normal lung matrix stiffness varies from 0.5 to 15 kPA, depending on the measurement within bronchi, vessels, and parenchyma [9,10]. At the cell-matrix level, collagen type I has been shown to be at least two orders of magnitude stiffer than elastin [11]. It has been suggested that these local differences, on a nano and micro scale, of the lung ECM could be important in regulating the spatial distribution, differentiation, and function of cells [12]. Of all the lung ECM fibrous proteins, collagen-I is the most abundant within the lung, and its expression is further increased in many fibrotic diseases, such as asthma, chronic obstructive pulmonary disease (COPD), and idiopathic pulmonary fibrosis (IPF) [13]. Collagen type I is, therefore, the most studied to influence the stiffness of the lung. Within the lung interstitium, fibroblasts are the most common cell type and a major cell type responsible for ECM production and regulation of normal ECM turnover by protein degradation [14]. The stiffness of the ECM has been shown to regulate the activation and fibrogenic properties of lung fibroblasts [15]. Alterations in the composition and/or modulation of stiffness of the lung ECM microenvironment in injured or disease lungs, therefore, has the potential to modify fibroblast functions and potentially drive tissue fibrosis, inflammation, and disease progression. It is now accepted that, in addition to immune cells, the structural cells of the lung, including lung fibroblasts, may also play a role in the initiation and maintenance of chronic lung inflammation through the production of pro-inflammatory cytokines and growth factors [16,17,18,19].

Currently, the investigation of fibroblast functions is largely based on studies on homogenous populations of cells cultured on flat substrates, also referred to as 2-dimensional (2D) culture. However, 2D cell cultures do not adequately represent the functions of 3D tissues that have extensive cell–cell and cell–matrix interactions, as well as markedly different diffusion or transport conditions. More recently, the complex interaction of fibroblasts within the lung ECM microenvironment has been modelled through the use of 3D scaffolds formed from collagen, Matrigel, or synthetic polymers polyethylene glycol (PEG) [20,21]. Studies with fibroblasts seeded into 3D scaffolds have shown that fibroblast contraction of collagen is important for fiber organization [22]. However, an important biological parameter that is not captured in static 3D collagen gels is the dynamic mechanics of the breathing lung, which are also altered in many diseases as a result of ECM and cellular changes [23]. Thus, there is a paucity of data on how a strain in a 3D ECM mechanodynamic environment influences fibroblast function. In this study, we used the Flexcell tissue train system to apply a mechanical strain to 3D collagen-I gels to model the dynamic environment that fibroblasts are exposed to within the normal breathing lung. We found that exposing lung fibroblasts to uniaxial mechanical strain and varying degrees of tissue stiffness caused alterations in cell morphology, cytoskeleton proteins, cell death, and the release of pro-inflammatory mediators (IL-6 and IL-8). This dynamic assay enables researchers to consider the assessment of the mechanodynamic nature of the in vivo lung ECM environment in disease-relevant models and the potential of mechano-pharmacology to identify therapeutic targets for treatment.

## 2. Materials and Methods

### 2.1. Fibroblast Cell Culture

Human fetal lung (HFL1) fibroblasts were commercially obtained (CCL-153, ATCC, Manassas, VA, USA) and cultured in Dulbecco’s Modified Eagle Medium (DMEM; Invitrogen, Burlington, ON, Canada), supplemented with 1× penicillin-streptomycin and 10% fetal calf serum (FCS) and incubated at 37 °C in humidified 5% CO_2_ atmosphere. When cells were confluent, they were harvested with 10% Trypsin-EDTA (Lonza, Bio Whittaker, Walkersville, MD, USA), stained with trypan blue and counted with the Countess™ II FL automated cell counter (Invitrogen, Burlington, ON, Canada), to determine the total number of viable cells. Cells were re-suspended in DMEM 10% FCS for experiments.

### 2.2. Collagen-I 2D and 3D ECM Environments

**Figure 1:**
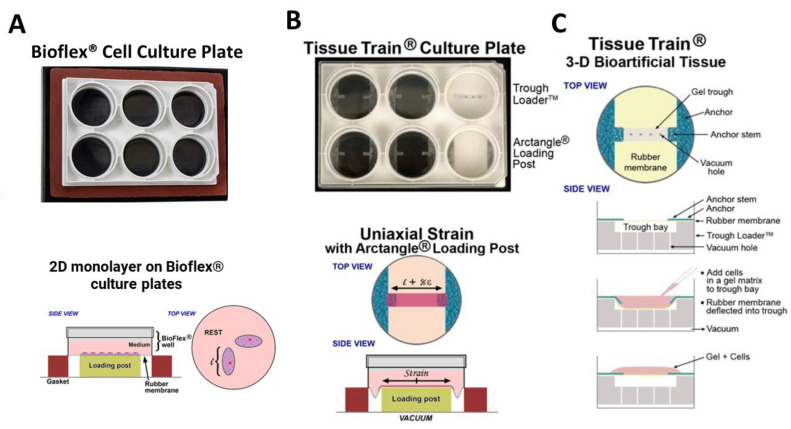
Flexcell Bioflex and Tissue Train models. (**A**) Image of a 6-well bioflex 6-well culture plate with a flexible membrane bottom and a schematic showing a 2D monolayer of cells grown in the plate. (**B**) Image of a 6-well tissue train culture plate placed on a loading post with the groove in which 3D collagen gels are made. A schematic showing the application of uniaxial strain on a linear fibroblast-seeded 3D collagen gel through the application of vacuum pressure underneath the loading post. (**C**) A schematic showing the production of fibroblast-seeded 3D linear gels in the Tissue train system before the application of strain. Adapted from Garvin et al. [24] and Flexcellint^®^.

For 2D cell culture experiments, HFL1 fibroblasts were seeded at a density of 100,000 cells in 3 mL DMEM 10% FCS per well on commercially available 6-well collagen-1-coated BioFlex^®^ plates (BF-3001C-Case, Flexcell, Burlington, NC, USA), and cultured for 24 h before experiments (Figure 1A). For 3D models, HFL1-cells were seeded in collagen-1 gels made with linear tissue constructs by using flexible bottomed 6-well Flexcell^®^ Tissue Train^®^ culture plates (TT-4001C, Flexcell, Burlington, NC, USA). The culture plates were placed on a 35 mm trough loader insert with a 25 × 3 × 3 mm^3^ linear groove containing four equally spaced 1-mm-diameter holes, through which a maximum vacuum of −90 kPa is applied to deform the flexible membrane of the culture plate [24]. The flexible membrane of the culture plate deforms and takes the shape of the groove under vacuum pressure (Figure 1B,C). Three-dimensional cell culture models were established by making collagen-1 gels using the standardized commercially available Collagel^®^ assay (COLKIT-100A, Flexcell, Burlington, NC, USA). This involved combining the components of the Collagel^®^ assay kit (Collagel^®^ (type 1 collagen), as well as reagent A (5× Minimum Essential Medium (MEM)), B (fetal bovine serum), C (1 M HEPES), and D (0.1 M NaOH)) in the volumes and ratios detailed in Table 1 according to the manufacturer’s instructions [24,25]. Furthermore, 50,000 HFL1 fibroblasts were resuspended per 200 μL of the Collagel mixture and carefully pipetted into the 25 × 3 × 3 mm^3^ linear groove of the Flexcell^®^ Tissue Train^®^ culture plate. The Tissue Train culture plate has foam anchors which secures the 3D linear construct in place. Linear HFL1 fibroblast-seeded collagen-I gels were formed after polymerization when incubated for 2 h in standard conditions (37 °C in humidified 5% CO2 atmosphere). After polymerization, 3 mL of DMEM 10% FCS was placed on the gels and incubated in standard conditions for 24 h. Depending on whether lung tissue repair occurs in healthy or abnormal (e.g., tissue with edema or scarring) in vivo environment, fibroblasts may encounter high or low collagen-1 concentrations [26]. To mimic the normal collagen concentration in the in vivo ECM, collagen-I gels were diluted to 0.4 mg/mL with 1× MEM [25], and a concentration of 2.0 mg/mL of collagen-I was used to mimic a high collagen-I concentration in vivo environment [25]. For all experiments, 3 technical replicates were established for 6 independent experimental conditions.

### 2.3. Mechanical Strain Experiments

After a 24-h incubation of HFL1-seeded collagen-I gels, the DMEM 10% FCS medium was replaced with DMEM containing 1% FCS. HFL1 fibroblast-seeded collagen-I gels were either left in the incubator without strain or were strained for 48 h with a uniaxial cyclic strain at an amplitude of 1% and at a frequency of 0.2 Hz to mimic the average normal breathing rate of 12 breaths/minute. The strain was experienced by all parts of the linear 3D construct equally through the vacuum applied to the flexible-bottom tissue culture train. This is because the flexible-bottom tissue culture plate placed on a trough loader insert of 35 mm completely fills the space underneath the 6-well tissue culture place with four equally spaced 1-mm-diameter holes. An amplitude of 1% was chosen since Garvin and colleagues, who developed the linear tissue construct, showed that higher amplitudes of more than 1% may cause matrix failure with magnitude-dependent experiments on linear tissue train constructs [24]. Again, a 1% strain falls in the range of the normal strain amplitudes experienced in the lung sub-epithelial space [27]. In addition to this, for kinetic experiments, HFL1 fibroblast-seeded collagen-I gels were strained for 5 and 30 min, 1 h, 3 h, and 48 h.

### 2.4. Immunofluorescence Imaging

The flexible rubber-bottom membrane of the Bioflex 2D plates were carefully cut for the staining of 2D-cultured HFL1s. HFL1s on the 2D membranes and in the various collagen-I models were fixed with 4% of paraformaldehyde for 20 min before being washed and permeabilized with 0.2% Triton X-100 for 10 min. Non-specific binding of proteins was blocked by incubating 2D membranes and collagen-I gels with 1% Horse serum for 1 h. An antibody against the cytoskeletal protein non-muscle myosin IIB (ab684, Abcam mouse anti-NMMIIB, Waltham, USA) diluted in 0.2% Triton X-100 and 1% horse serum was then incubated with the sample overnight at 4 °C. Two-dimensional and three-dimensional cultures were also stained with an Alexa Fluor 488 Phalloidin (A12379, Thermofisher, Waltham, MA, USA) to stain the F-actin cytoskeleton. Cultures were then washed again in PBS and incubated with the 4′,6-diamidino-2-phenylindole nuclei stain (DAPI, 32670-25MG-F, Sigma, Oakville, ON, Canada) for 10 min. The 2D membranes and gels were then mounted on glass slides and imaged with a confocal microscope, as previously described [22]. HFLs were also stained with a mouse IgG as a negative control for NMIIB which showed no staining (See Supplemental Figure S1). Images were acquired with a Zeiss LSM880 (Carl-Zeiss-Straße 22, 73,447 Oberkochen, Germany) inverted confocal microscope, equipped with an Airyscan detector. The imaging parameters were kept the same for all experiments to eliminate varying levels of pixel brightness, differences in diffraction between the two sample types, and differences in background fluorescence. To analyze the intensity of immunofluorescent staining, ImageProPlus was used to count positive pixels for non-muscle myosin IIB and F-actin staining in 20X images of three regions of interest (ROI) on each 2D membrane and collagen gel. Pixels for all cell numbers in each image ranging from 5–70 cells were counted. Stain intensity was then expressed as the percentage positive pixel of total pixels. For the kinetic experiments, the CellProfiler^®^ software was used to employ a supervised machine learning approach using a training set of 200 cells pulled from all time points. This allowed the training of the software to segment pixels as nuclear, cytoplasmic, or background in each single HFL1 fibroblasts in different ROIs at various time points. The segmented cells in bulk images per time point were then classified as non-muscle myosin IIB or actin positive cells through CellProfiler^®^, and were presented as a TSNE plot, visualizing every single cell within the 3D culture over the different time points and the expression of non-muscle myosin IIB.

### 2.5. ELISA and Lactate Dehydrogenase Assays

The concentrations of IL-8 and IL-6 released by HFL1 fibroblasts were measured in the supernatant medium using commercial ELISAs (R&D Systems, Minneapolis, MN, USA), according to the manufacturers’ instructions. The concentrations of IL-8 and IL-6 were normalized to the average total cell counts when comparing between 2D and 3D models. The standard curve for IL-8 ranged from 2000 pg/mL to 31.3 pg/mL while that of IL-6 ranged from 600 pg/mL to 9.38 pg/mL. A four-parameter logistic curve fit was generated for both ELISAs. The concentrations of diluted samples read from the standard curve fit were then multiplied by their dilution factors. To measure the cell death of HFL1 fibroblasts, the release of lactate dehydrogenase in the supernatant medium was measured using a commercially available LDH assay kit (Thermofisher, Waltham, MA, USA), according to the manufacturer’s instructions.

### 2.6. Statistics

The differences between paired observations were assessed with paired *t*-tests and 1-way ANOVA with post-hoc Tukey test. Unpaired observations were assessed with an unpaired t-test. Interactions between strain and collagen-I concentrations or strain and time were assessed with a 2-way ANOVA with a Sidak post-hoc test. All tests were completed in GraphPad version 9, and *p* < 0.05 was considered statistically significant.

## 3. Results

### 3.1. Effect of 2D and 3D Collagen-I Models on Lung Fibroblast Phenotype and Function

**Figure 2:**
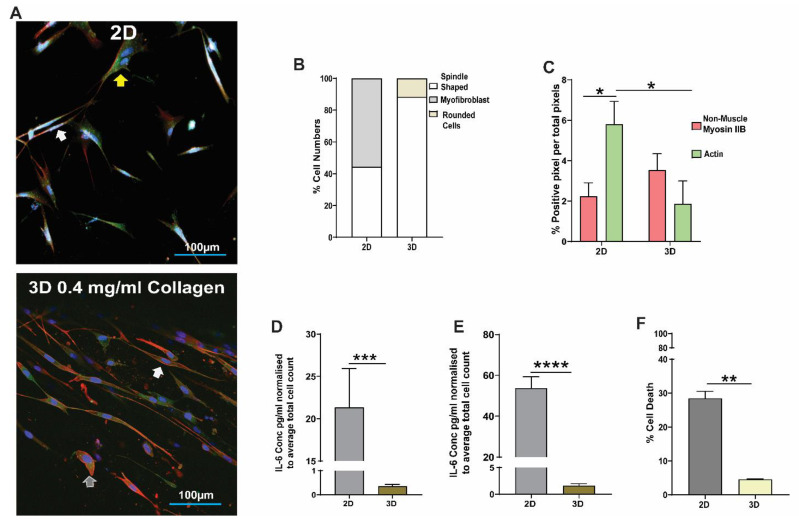
The effect of 2D and 3D collagen-I models on lung fibroblast phenotype and function. Human fetal lung 1 (HFL1) fibroblasts were cultured in a 2D monolayer on collagen-coated flexible Bioflex plates or embedded in 3D collagen-1 gels and cultured for 24 h. Representative confocal image of HFL1s in (**A**) 2D monolayer and (**B**) 3D collagen-1 gels after culturing for 24 h and staining for non-muscle myosin IIB (red) and F-actin (green). (**C**) Percentage cell numbers of spindle shaped, myofibroblast, and rounded HFL1 fibroblasts in 2D and 3D culture models. (**D**) Percentage positive pixel count per total number of pixels in images of 2D and 3D culture models. The concentration of (**E**) IL-6 and (**F**) IL-8 cytokines released from HFL1 fibroblasts in 2D and 3D culture models normalized to total cell counts. The percentage cell viability of HFL1 fibroblasts in 2D and 3D culture models. Mean ± SEM indicated for 3 technical replicates, *n* = 6. * *p* < 0.05, ** *p* < 0.01, *** *p* < 0.001, **** *p* < 0.0001.

Collagen-I at a concentration of 0.4 mg/mL was shown to mimic the normal collagen-I ECM environment in healthy tissue in vivo [25]. We compared the response of HFL1 fibroblasts to collagen-I on a 2D collagen-1-coated monolayer versus a 3D collagen-I gel (0.4 mg/mL). Figure 2A shows representative images comparing HFL1 fibroblasts on 2D collagen-I-coated plates and 3D 0.4 mg/mL collagen-I gels stained for DNA (blue), F-actin (green), and non-muscle myosin IIB (red). On 2D collagen-I-coated monolayers, 48% of HFL1 cells had a spindle-shaped morphology (white arrow) and 52% had a myofibroblast morphology (yellow arrow) (Figure 2B). In comparison, in 3D collagen-I cultures, 88% of HFL1 cells had a spindle-cell morphology (white arrow) and 12% had a ‘rounded’ cell morphology with loss of dendritic attachments (ash arrow) (Figure 2B). When we assessed the cytoskeleton composition, HFL1 fibroblasts within the 2D collagen-I monolayer were predominantly positive for the cytoskeletal protein F-actin compared to HFL1 fibroblasts in the 3D collagen-I gels which were mostly non-muscle myosin IIB positive cells (Figure 2C, *p* < 0.05). We next assessed the release of inflammatory cytokines and found that HFL1 fibroblasts cultured on 2D collagen-I monolayers released increased concentrations of IL-6 (Figure 2D, *p* < 0.01) and IL-8 (Figure 2E, *p* < 0.01), compared to HFL1 fibroblasts seeded in 3D collagen-I gels. Lastly, we found a significant increase in the percentage of cell death in HFL1 fibroblasts cultured on 2D collagen-I monolayers compared to 3D collagen-I gels (Figure 2F, *p* < 0.01).

### 3.2. Response of Lung Fibroblasts to a High Concentration Collagen-I 3D Microenvironment

**Figure 3:**
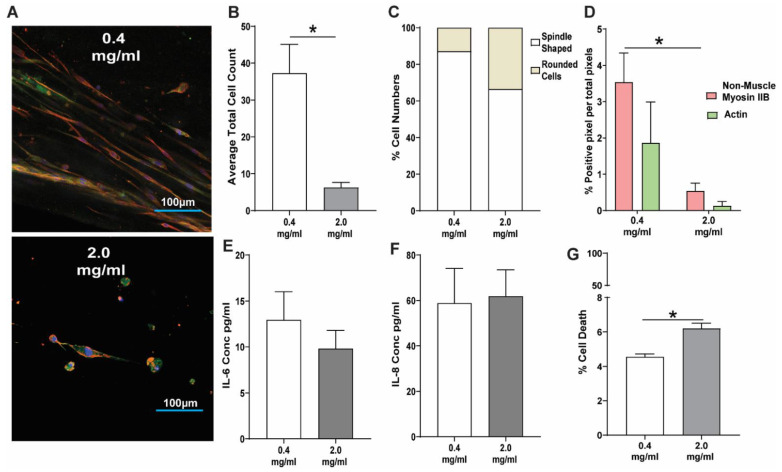
The response of lung fibroblasts to fibrotic collagen-I 3D microenvironment. Human fetal lung 1 (HFL1) fibroblasts were embedded in 3D collagen type-I gels of 0.4 mg/mL and 2.0 mg/mL concentration and cultured for 24 h. Representative confocal image of HFL1s in (**A**) 0.4 mg/mL and (**B**) 2.0 mg/mL collagen-1 gels after culturing for 24 h and staining for non-muscle myosin IIB (red) and F-actin (green). (**C**) Average total cell counts in 0.4 mg/mL and 2.0 mg/mL collagen-I gels. (**D**) Percentage cell numbers of spindle and rounded shaped cells in 0.4 mg/mL and 2.0 mg/mL collagen-I gels. (**E**) Percentage positive pixel count per total number of pixels in images of 0.4 mg/mL and 2.0 mg/mL collagen-I gels. The concentration of (**F**) IL-6 and (**G**) IL-8 cytokines released from HFL1 fibroblasts in 0.4 mg/mL and 2.0 mg/mL collagen-1 gels. (**G**) Percentage cell viability of HFL fibroblasts in 0.4 mg/mL and 2.0 mg/mL collagen-I gels. Mean ± SEM indicated for 3 technical replicates, *n* = 6. * *p* < 0.05.

Figure 3A shows representative images comparing HFL1 fibroblasts in a low-concentration collagen-I environment (0.4 mg/mL) (25), compared to a high-concentration and stiffer (2.0 mg/mL) collagen-I environment (24) stained for DNA (blue), F-actin (green), and non-muscle myosin IIB (red). Following 48 h of culture, the high-concentration collagen-I 3D model (2.0 mg/mL) had a decreased HFL1 fibroblast cell count compared to HFL1 fibroblasts seeded in a low-concentration 0.4 mg/mL collagen-I microenvironment (Figure 3B). For both concentrations of 0.4 and 2.0 mg/mL collagen-I, there was no change in the morphology of cells with 88% and 68% of HFL1 fibroblasts, respectively, with a spindle-shaped morphology (Figure 3C). Additionally, the percentage of positive pixels for non-muscle myosin IIB or F-Actin was not altered in the two collagen-I concentrations, despite the total staining being decreased in the 2.0 mg/mL collagen-I gels due to a decreased number of total cells (Figure 3D). When the release of inflammatory cytokines was analyzed, we found no differences in the release of IL-6 (Figure 3E) and IL-8 (Figure 3F) in HFL1 fibroblasts, irrespective of collagen-I concentration. However, we did observe a small increase in the percentage of cell death in HFL1 fibroblasts cultured in the ‘fibrotic’ collagen-I gels compared to the ‘normal’ collagen-I gel (Figure 3G, *p* < 0.05).

### 3.3. Response of Lung Fibroblasts to Mechanical Strain within a 3D Collagen-I Microenvironment

**Figure 4:**
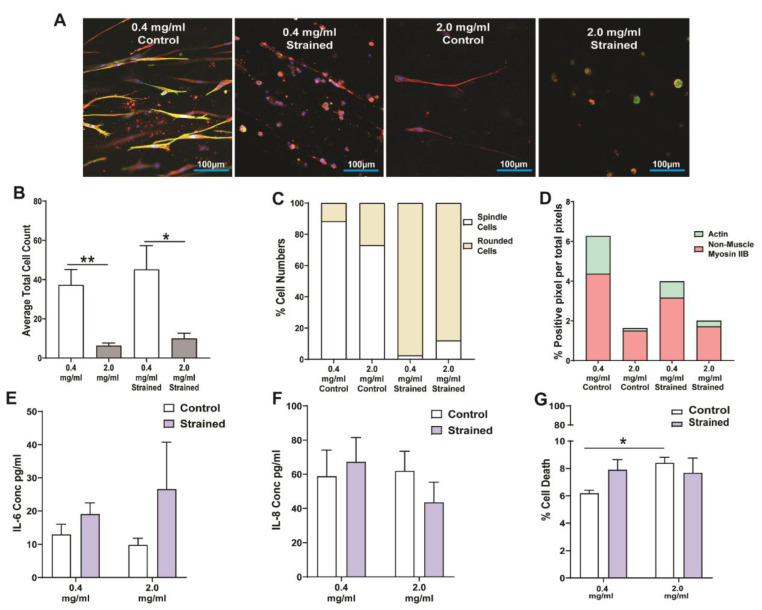
Response of lung fibroblasts to mechanical strain within a 3D collagen-I microenvironment. Human fetal lung 1 (HFL1) fibroblasts were embedded in 3D collagen-1 gels of 0.4 mg/mL and 2.0 mg/mL concentrations and cultured for 24 h. The HFL1 fibroblast-seeded collagen gels were then left alone or mechanically strained for 48 h at a 1% amplitude and a frequency of 0.2 Hz. (**A**) Representative confocal images of HFL1 fibroblasts in 0.4 mg/mL and 2.0 mg/mL collagen-1 gels stained for non-muscle myosin IIB (red) and F-actin (green) after mechanical strain experiments. (**B**) Average total cell counts in 0.4 mg/mL and 2.0 mg/mL collagen-1 gels after strain or no strain for 48 h. (**C**) Percentage cell numbers of spindle and rounded shaped cells in 0.4 mg/mL and 2.0 mg/mL collagen-I gels after strain or no strain for 48 h. (**D**) Percentage positive pixel count per total number of pixels in images of 0.4 mg/mL and 2.0 mg/mL collagen-I gels after strain or no strain for 48 h. (**E**) Percentage positive pixel count per total number of pixels in images of 0.4 mg/mL and 2.0 mg/mL collagen-I gels after strain or no strain for 48 h. The concentration of (**F**) IL-6 and (**G**) IL-8 cytokines released from HFL1 fibroblasts in 0.4 mg/mL and 2.0 mg/mL collagen-1 gels after strain or no strain for 48 h. (**G**) The percentage of cell viability of HFL fibroblasts in 0.4 mg/mL and 2.0 mg/mL collagen-I gels after strain or no strain for 48 h. Mean ± SEM indicated for 3 technical replicates, *n* = 6. * *p* < 0.05, and ** *p* < 0.01.

We applied a strain amplitude of 1% at a normal breathing frequency of 0.2 Hz for 48 h to investigate how HFL1 fibroblasts respond to a low- and high-concentration collagen-1 ECM environment within the dynamic breathing lung. Figure 4A shows representative images comparing HFL1 fibroblasts in a low-concentration collagen-I environment (0.4 mg/mL) compared to a high-concentration collagen-I environment (2.0 mg/mL) with and without 1% uniaxial strain (0.2 Hz) stained for DNA (blue), F-actin (green), and non-muscle myosin IIB (red). While 2.0 mg/mL collagen-1 gels had fewer total HFL1 fibroblasts compared to 0.4 mg/mL collagen-I gels, the addition of 1% strain at a frequency of 0.2 Hz did not alter the cell number within the 3D cultures (Figure 4B). However, when we assessed the morphology of HFL1 fibroblasts, we found that the 48 h exposure of 1% strain (0.2 Hz) to the HFL1-seeded linear tissue train gel caused a dramatic loss of fibroblastic dendritic extensions in HFL1 fibroblasts that led to an increased number of rounded fibroblasts, regardless of the collagen-I gel concentration (Figure 4C). Assessment of the cytoskeletal proteins showed that mechanical strain did not affect the predominance of the expression of non-muscle myosin IIB expression, in either the 0.4 or 2.0 mg/mL 3D gel collagen-I gels (Figure 4D, *p* < 0.05). When we assessed the effect of mechanical strain on inflammatory mediator release and cellular cytotoxicity, we found no effect on the release of IL-6 (Figure 4E) and IL-8 (Figure 4F), or on the percentage of cell death (Figure 4G).

### 3.4. Kinetics of Lung Fibroblast Cytoskeleton and Morphological Alterations Following Mechanical Strain

**Figure 5:**
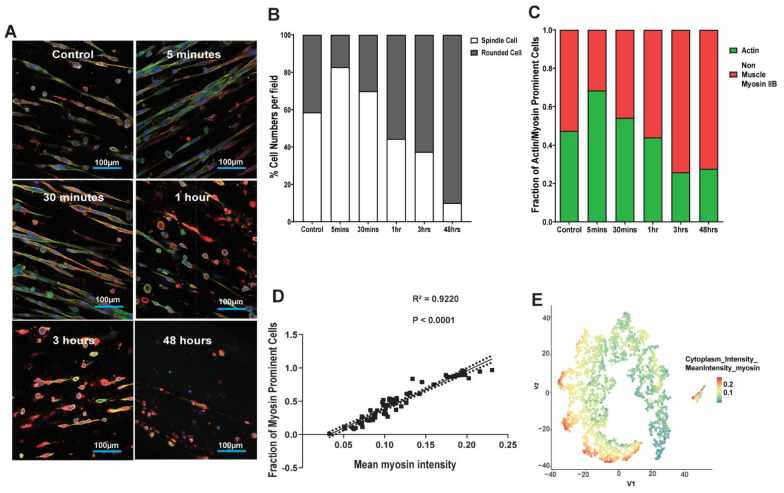
Kinetics of lung fibroblast cytoskeleton and morphological alterations following mechanical strain. Human fetal lung 1 (HFL1) fibroblasts were embedded in 3D collagen-1 gels of 2.0 mg/mL concentrations and cultured for 24 h. The HFL1 fibroblast-seeded collagen gels were then left alone or mechanically strained for 48 h at a 1% amplitude and a frequency of 0.2 Hz. Gels were collected after 0, 5, and 30 min, and 1, 3, and 48 h, following the stain protocol. (**A**) Representative confocal images of HFL1 fibroblasts in 2.0 mg/mL collagen-1 gels stained for non-muscle myosin IIB (red) and F-actin (green) after mechanical strain experiments at different time points. (**B**) Percentage cell numbers of spindle and rounded shaped cells at different time points in 2.0 mg/mL collagen-1 gels after being strained at different time points. (**C**) Percentage positive pixel count per total number of pixels in images of 2.0 mg/mL collagen-1 gels after being strained at different time points. (**D**) The correlation between the fraction of myosin prominent cells and the mean myosin intensity in images of 2.0 mg/mL collagen-1 gels after being strained at different time points. (**E**) TSNE-plot visualization of each single cell with the 2.0 mg/mL collagen-1 gels after being strained at different time points. Mean stacked bar graphs are shown for 3 technical replicates, *n* = 6 in (**B**,**C**).

As there was a clear difference in the effect of mechanical strain on collagen-I gel-seeded-HFL1 fibroblasts during the 48 h experiment, we sought to determine if the change in cell morphology was due to the duration of strain in our experiments. Further, as the effect of strain was not different between the 0.4 and 2.0 mg/mL collagen-I concentrations, we chose to only assess the kinetics of HFL1 fibroblasts cell morphology in the 2.0 mg/mL collagen-I gels. Figure 5A shows the representative images of 2.0 mg/mL collagen-I gels seeded with HFL1 fibroblasts at 0, 5, and 30 min, and at 1 h, 3 and 48 h, following mechanical strain. Following 30 min of 1% strain (0.2 Hz), the number of spindle-shaped HFL1 fibroblasts gradually decreased with a corresponding increase in rounded cells up to the 48 h time point (Figure 5B, *p* < 0.05). In addition to the loss of dendritic attachments, we found a corresponding decrease in F-actin staining, coupled with increasing staining of non-muscle myosin IIB from 30 min which then plateaued between 3 and 48 h (Figure 5C, *p* < 0.05). This observation was confirmed by a strong positive correlation between the fraction of non-muscle myosin IIB positive cells and the mean intensity of non-muscle myosin IIB staining in HFL1 fibroblasts from all the time points (Figure 5D). Figure 5E visualizes each single cell within the 3D culture and demonstrates that, over the time course of the experiment, each of the cells had increased expression of non-muscle myosin IIB.

## 4. Discussion

This study demonstrates that human fetal lung fibroblasts grown in a 3D compared to 2D collagen type-I environment do not form cells with a myofibroblast morphology, express less F-actin stress fibers, exhibit less cell death, and significantly produce less pro-inflammatory IL-6 and IL-8 cytokines. Exposure to mechanical strain to mimic normal breathing (0.2 Hz) led to the rounding of HFL1 fibroblasts due to the loss of dendritic extensions, as well as the loss of F-actin stress fibers within the cell cytoskeleton, but did not influence pro-inflammatory cytokine production or cell death. In summary, the demonstrated 3D mechanical model of the collagen-I lung environment provides a sensitive and biologically relevant tool for investigating different fibrotic and pro-inflammatory events involving lung fibroblasts.

Fibroblasts are the primary cells responsible for collagen and elastin synthesis, and the organization of the ECM components. During wound healing and tissue remodeling, fibroblasts are indispensable in determining how a wound will heal or tissue will remodel. To date, the majority of studies have focused on wound closure in dermal models. Previously, wound contraction was initially thought to be caused by the new deposition of collagen fibers. However, in 1956, Abercrombie et al. demonstrated that contraction and remodeling of granulation tissue were more important for wound closure than the formation of de novo collagen fibers [28]. It has since been postulated that specialized fibroblasts within granulation tissue transition into myofibroblasts, and that these cells are responsible for ECM contraction and wound closure [29]. In addition, it has also been proposed that fibroblasts within granulation tissue exert traction on the surrounding ECM via cell locomotion forces, which realign the collagen fibrils closing the wound [30]. The fibroblast cytoskeleton consists of three types of filaments (actin microfilaments, intermediate filaments, and microtubules), with actin filaments being considered the most significant for modulating the mechanical properties of cells [31]. Compared to fibroblasts, myofibroblasts are rich in cytoplasmic actin-stress fibers, and the reorganization of the actin filaments leads to changes in the cell morphology [23]. Previously studies by Grinnell et al. demonstrated that fibroblasts in a tethered collagen matrix form prominent stress fibers, whereas fibroblasts seeded in a free-floating collagen matrix maintain a spindle-like fibroblast morphology [32]. Our study demonstrates that, when fibroblasts are seeded in a tethered 3D compared to 2D collagen type-I gel, lung fibroblasts do not exhibit a myofibroblast morphology and express less F-actin stress fibers assessed using phalloidin staining, which intercalates into polymerized F-actin. Following wound repair, myofibroblasts are supposed to undergo programmed cell death (apoptosis) [33]. It is, therefore, interesting that when there are no cells with a myofibroblast phenotype in the 3D-tethered collagen-I gels, there is also a significant reduction in cell death (5%) compared to the 2D collagen-I cultures, whereby 50% of cells have a myofibroblast morphology with a 30% cell death. These data highlight that a 3D ECM environment, compared to a 2D one, is very important for understanding lung repair and tissue remodeling, especially with regards to the role and morphology of lung fibroblasts, cell cytoskeleton, and cell death.

We found that when HLF1 fibroblasts were exposed to high collagen-I concentrations (2.0 mg/mL), the fibroblasts lost their dendritic extensions, and the expression of F-actin was decreased. Previous studies have investigated the effect of collagen-I concentrations on fibroblast function. In support of our findings, Plant et al. previously found that fibroblast cells spread poorly on 2D monolayers in high concentrations of collagen-I (750 and 250 μg/mL) [34]. In 1979, Bell et al., using a 3D floating collagen gel and human fibroblast cells, found an increased concentration of collagen-I (220 μg/mL, 360 μg/mL, and 570 μg/mL), which decreased the diameter (mm) of fibroblast cells over 10 days [20]. More recently, Grace et al. previously showed that, compared to collagen-I gels, collagen-I gels seeded with PEG, which results in thicker collagen fibers and stiffer gels, leads to the rounding of fibroblasts [35]. Together, these data, therefore, support the current notion that a ‘fibrotic’ collagen environment affects the ability of fibroblasts to form dendritic extensions and effectively remodel the ECM environment. In contrast to these data, stiffening and remodeling of the ECM environment in 2D models have been shown to lead to an increased area of cell spread, the formation of stress fiber and focal adhesion complexes, and increased proliferation in fibroblasts [36]. With such contrasting results between 2D or 3D collagen-I models, it supports much further work to understand the effects of ECM stiffness on fibroblast function in 3D models that mimic the in vivo setting.

Inflammation plays a central role in many chronic lung diseases, such as asthma, COPD, and IPF, with each disease having its unique inflammatory profile and anatomical site of disease. Immune cells recruited into the lung are regarded as key effector cells of disease through the production of various pro-inflammatory cytokines. However, in diseases associated with chronic inflammation, it is now accepted that the structural cells, including epithelium, fibroblasts, and endothelium, may also play an important role in the maintenance of chronic lung inflammation. In this study, we demonstrate that the culture of HFL1 fibroblasts in a 3D versus 2D collagen-I environment results in almost a complete loss of pro-inflammatory IL-6 and IL-8 cytokine expression. These findings are in line with Htwe et al., who looked at nuclear factor NF-kB signaling in 2D and 3D culture-systems and found a significant reduction in NF-kB signaling in 3D cultures [37]. While no cytokine expression was measured in the previous study by Htwe et al., NF-kB is known to induce IL-6 and IL-8 expression from several cell types, including fibroblasts. IL-6 is an acute response inflammatory cytokine and IL-8 is a neutrophil chemoattractant, and both cytokines have been shown to be elevated in many chronic inflammatory lung diseases. Future studies will be required to understand if only NF-Kb-derived cytokines are influenced by the collagen environment or other cytokines and growth factors. In this study, we identified that the concentration of collagen-I in a 0.4 or 2.0 mg/mL gel and/or mechanical strain to mimic normal breathing did not influence IL-6 and IL-8 production by HLF cells. Together, these data have important implications for the model used to study the role of fibroblasts in regards to inflammation, especially when studying cells derived from patients with inflammatory associated diseases. While these data indicate differences in the inflammatory mediator release of IL-6 and IL-8 from fibroblasts in 3D models, there are limitations to note with this experiment. While both the 2D and 3D models used collagen I, the cells within the 3D model are, however, encapsulated by collagen-I; therefore, it is possible that IL-6 and IL-8 could be sequestered within the collagen-I gel. However, there is no literature to date which indicates that IL-6 and IL-8 bind to collagen-I. Further, IL-6, an acute phase inflammatory cytokine, and IL-8, a neutrophil chemoattractant, are both released from injured or infected tissues into the blood stream to initiate inflammatory cell recruitment, activation, and responses from other organs, such as α1-antitrypsin release from the liver. Therefore, both IL-6 and IL-8 can be found in high levels in the blood during infection or inflammation; therefore, we assume that the soluble IL-6 and IL-8 measured from the model account for the total protein, but future experiments looking at IL-6 and IL-8 RNA production will be important to confirm this.

Previous studies that assessed how mechanical strain affects lung fibroblast behavior have used 2D monolayer models and high strain amplitudes (up to 30%) to mimic the strain in an airway during an asthmatic episode [27,38]. In this study, we chose to develop our mechanical 3D model with a lower strain amplitude of 1%, which has been reported to be experienced by the basement membrane in the developing lung of the fetus [27]. We also used a normal breathing frequency of 0.2 Hz and conducted the experiment for 48 h to carefully tease out the long-term effects of mechanical strain on HFL1 fibroblast functions within a 3D collagen-I matrix. When we applied mechanical strain to both a ‘low’ and ‘high’ collagen-I environment, we demonstrate that a 1% strain at a normal breathing frequency of 0.2 Hz had a significant effect on fibroblast morphology with significant cell rounding and loss of dendritic extensions. Manuyakorn et al. used 2D plates to find that the effect of cyclical mechanical strain at the maximal amplitude of 30% (to mimic an asthmatic episode) resulted in fibroblasts with an elongated cell morphology at the periphery of the well (high strain) and a stellate morphology in the middle of the well (minimal strain) [38]. Further, Manuyakorn et al. also found that increasing levels of strain in the 2D model led to the loss of stellate morphology and dendritic extensions [38]. Importantly, when we looked at the kinetics of the alterations in the fibroblast structure and cell cytoskeleton, we found that the loss of the spindle cell morphology was associated with the loss of F-actin, and an increase in the expression of non-muscle myosin IIB. This rearrangement of actin is supported by a study by Grymes et al., suggesting that any alteration in fibroblast morphology is directly related to the degree of actin polymerization [39]. Our work demonstrates the need for growing cells in a representative three-dimensional tissue environment if we are to understand the true effects of strain on biological tissues.

There are limitations to note in this study. Firstly, the study used HFL1 fibroblasts which, while a robust and commonly used model of lung fibroblasts, does not relate to potential differences in fibroblasts derived from the parenchyma of patients with chronic lung diseases, such as IPF or asthma. Secondly, while the model provides a 3D ECM environment that can be mechanically stretched, the use of collagen-I alone does not take into account the variation in cell responses that may occur when cells are seeded with a lung ECM containing collagen type I, II, or III, as well as fibronectin, laminins, and vitronectin. The biomechanical forces of ECM additionally influence the activation of growth factors. It has been shown that stiffer ECM in lung fibrosis provides a strong stimulant that exacerbates pro-fibrotic signaling in this microenvironment [40]. Future experiments using acellular scaffolds from normal and fibrotic lungs [41] or lung bioinks [42] may provide better 3D models on the lung ECM, to which a mechanical strain can be applied to better model the dynamics of the ECM lung environment in health and disease. Further, future work is also required to assess the effect of mechanical strain on collagen fiber organization by lung fibroblasts, which is possible with second harmonic imaging of collagen fibers. Such work will be important to understand how mechanical strain influences the structure of the ECM environment within the lung.

## 5. Conclusions

In conclusion, we found that exposing human fetal lung (HFL1) fibroblasts to uniaxial mechanical strain in 2D and 3D collagen-1 environments caused alterations in cell morphology, cytoskeleton proteins, and cell death, as well as the release of pro-inflammatory mediators IL-6 and IL-8. Further, alterations in collagen-I to mimic a low- (0.4 mg/mL) and high- (2.0 mg/mL) collagen concentration lung environment influenced fibroblast proliferation and cell phenotype, with the loss of F-actin and spindle-shape morphology. This study demonstrates that mechanical strain, in addition to ECM concentration and, therefore, stiffness, has a large effect on lung fibroblast morphology. Thus, future work is required to understand the mechano-dynamic environment, especially in the setting of fibrotic diseases, such as COPD and IPF, where both the ECM environment and lung mechanics are significantly altered.

## Figures and Tables

**Table 1:** Volumes of Collagel^®^ kit components, as standardized by Flexcellint^®^.

Reagent	Volume (µL) Ratios for 6-Well Tissue Train^®^ (200 µL/well)
A (5× MEM)	288 µL
B (Fetal Bovine Serum)	144 µL
C (1M Hepes)	36 µL
Collagel^®^ (Type 1 Collagen, 3 mg/mL in 0.01 MHCl)	1260 µL
D (0.1 M NaOH in 5× MEM)	72 µL

## Data Availability

There are no datasets attached with the paper.

## Supplementary Materials

The following are available online at https://www.mdpi.com/article/10.3390/cells11020185/s1, Figure S1: Human lung fibroblasts (HFLs) were stained with (A) DAPI (4′,6-diamidino-2-phenylindole) to identify nuclei and mouse IgG isotype control and rabbit anti-mouse IgG conjugated with Alexa 594 or (B) mouse IgG antibody for non-muscle myosin IIB and rabbit anti-mouse IgG con-jugated with Alexa 594. Both IgG isotype control and non-muscle myosin IIB had a concentration of 0.61mg/mL and were used at a 1 in 200 dilution. Figure S2: Human lung fibroblasts (HFLs) were seeded in 2D culture plates and stained with (A) (4′:6-diamidino-2-phenylindole) to identi-fy nuclei, (B) phalloidin to assess F-actin fibers, (C) non- muscle myosin IIB and (D) visualises the staining from all markers merged.
