# Supplementary material for: The Role of the Dynamic Lung Extracellular Matrix Environment on Fibroblast Morphology and Inflammation
Source: Cells. 2022 Jan 6;11(2):185. doi: 10.3390/cells11020185 (PMC8773771; doi:10.3390/cells11020185)
Supplement: Supplementary file 1 [file cells-11-00185-s001.zip › cells-1505531-supplementary.pdf]

SUPPLEME
NT

The role of the dynamic lung extracellular matrix environment on fibroblast morphology andinflammation

Tillie-Louise Hackett[1,2], Noamie T.R.F Vriesde[1], May AL-Fouadi[1,2], Leila Mostaco-Guidolin[3], Delaram Maftoun[1,2], Aileen Hsieh[1,2], Nicole Coxson[1,2], Kauna Usman[1,2], Don D. Sin[1,4], Steven Booth[1], Emmanuel T.Osei[1,5],*

1 Centre for Heart Lung Innovation, St. Paul's Hospital, Vancouver, BC, Canada

2 Department of Anesthesiology, Pharmacology and Therapeutics, University of British Columbia,Vancouver, BC, Canada

3 Department of Systems and computer engineering, Carleton University, Ottawa, ON, Canada

4 Department of Respiratory, University of British Columbia, Vancouver, BC, Canada

5 Department of Biology, University of British Columbia, Okanagan, BC, Canada

* Correspondence: emmanuel.osei@ubc.ca; UBC-Okanagan, Biology Department, 1177 ResearchRoad, SC 217, Kelowna BC, V1V1V7

A B

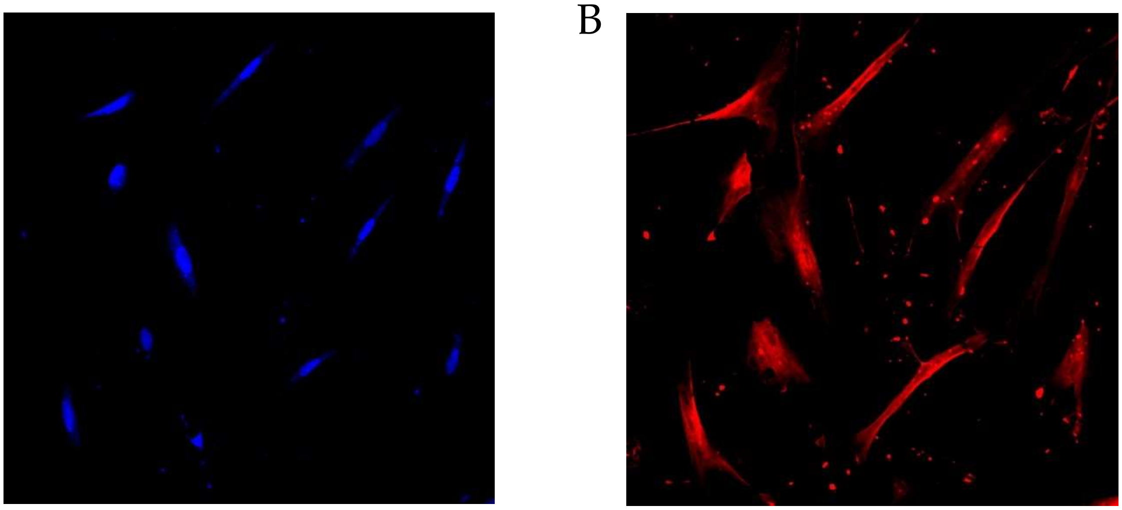

Supplemental Figure S1. Human lung fibroblasts (HFLs) were stained with (**A**) DAPI (4′,6-diamidino-2- phenylindole) to identify nuclei and mouse IgG isotype control and rabbit anti-mouse IgG conjugated with Alexa 594 or (**B**) mouse IgG antibody for non-muscle myosin IIB and rabbit anti-mouse IgG conjugated with Alexa 594. Both IgG isotype control and non-muscle myosin IIB had a concentration of0.61mg/ml and were used at a 1 in 200 dilution.

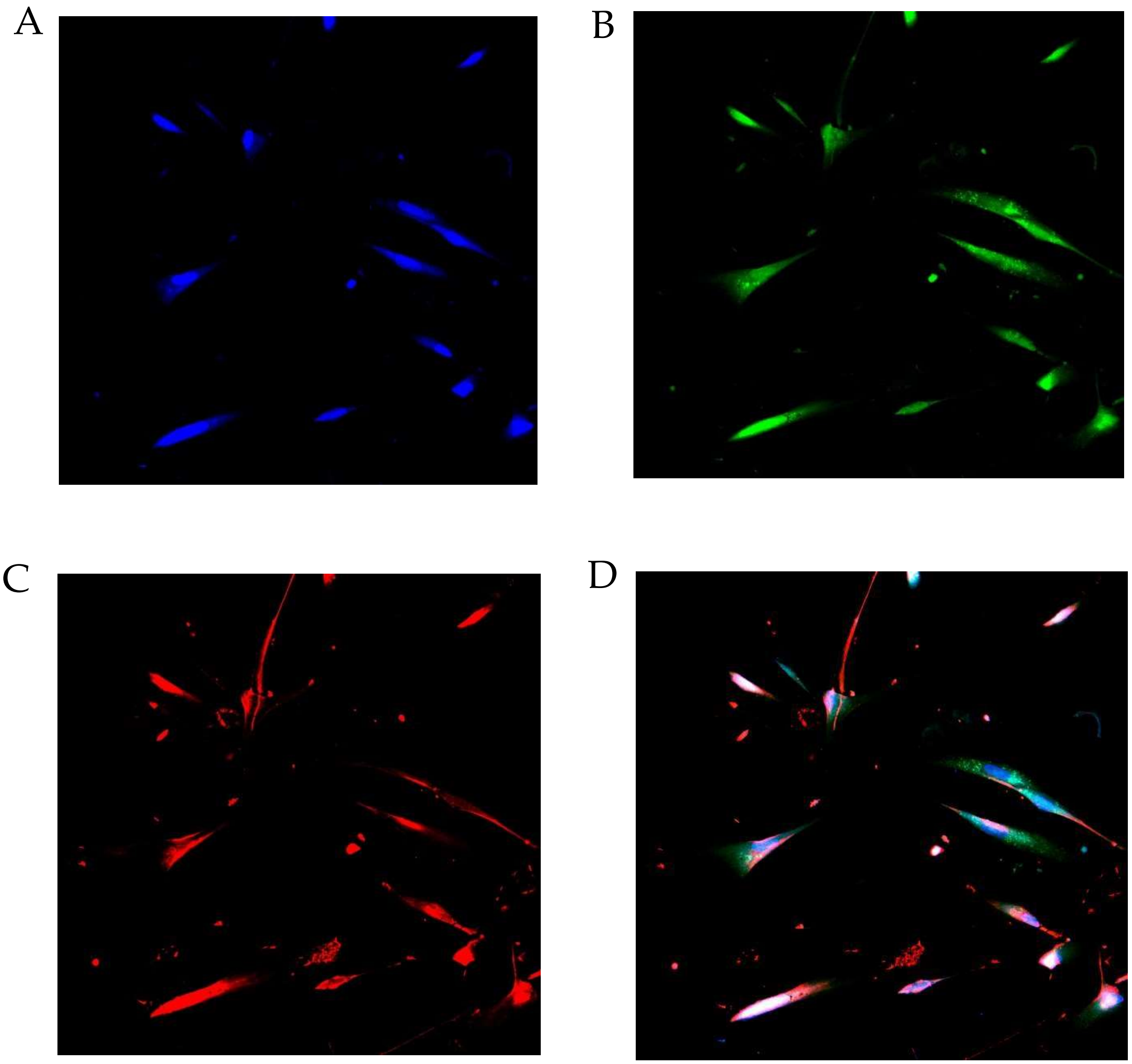

Supplemental Figure S2: Human lung fibroblasts (HFLs) were seeded in 2D culture plates and stained with (**A**)DAPI (4′,6-diamidino-2-phenylindole) to identify nuclei, (**B**) phalloidin to assess F-actin fibers, (**C**) non-muscle myosin IIB and (**D**) visualises the staining from all markers merged.
